# Developing Effective Salmonella-based Approaches to Treat Pancreatic Cancer

**DOI:** 10.4172/2165-7092.1000167

**Published:** 2016

**Authors:** Jeremy Chen, Don J. Diamond, Edwin R. Manuel

**Affiliations:** Department of Experimental Therapeutics, City of Hope, Duarte CA, USA

**Keywords:** Salmonella, Pancreatic cancer, Treatment, Pancreatic ductal adenocarcinoma, Tumor malignancies

## Commentary

Pancreatic ductal adenocarcinoma (PDAC) is predicted to have the second highest incidence of fatalities among all solid tumor malignancies worldwide. Despite three decades of research focused on treating PDAC, the five-year survival rate is less than 6%, thus leaving much room for improvement. Historically, PDAC has been difficult to treat due to the absence of early detection methods resulting in clinical disease that is significantly advanced and characterized by immunosuppression, an extreme form of fibrosis known as desmoplasia, and metastasis to vital organs [1]. Current chemotherapeutic combinations, such as gemcitabine with Abraxane, have been shown to extend patient survival; however, they only do so by two to three months [2]. The newly released irinotecan liposome injection tested in pancreatic cancer patients is, at best, equal in efficacy to gemcitabine with Abraxane [3-5]. The low efficacy and high toxicity of chemotherapy has lead to innovative new strategies using immunotherapeutic approaches for treatment of PDAC. For example, the whole pancreatic cancer cell vaccine expressing human macrophage-colony stimulating factor (GM-CSF), known as GVAX, combined with a Listeriabased vaccine expressing the PDAC antigen mesothelian (CRS-207) shows promise in extending survival evidenced by both pre-clinical and preliminary clinical data [6]. Furthermore, antibody therapies targeting CTLA-4 and PD-1 have shown great benefit toward enhancing antitumor immunity resulting in tumor regression and extension of survival in other solid tumor models [7]. Despite these successes, there is a growing consensus that a “multi-pronged” approach to induce anti-tumor immunity and simultaneously targeting immune suppression and desmoplasia will have the greatest effect in eliminating PDAC. However, balancing such aggressive approaches with minimal toxicity to the patient will prove to be an incredibly daunting task.

We first tested the hypothesis of targeting immune suppression to enhance tumor-specific responses in an aggressive, highly immunogenic murine model of melanoma [8]. We found that therapeutic vaccination alone using the tumor antigen survivin, which is over-expressed and enhances survival in melanoma cells, was unable to generate tumor-specific responses to control melanoma growth. However, when we administered, prior to vaccination, an attenuated Salmonella typhimurium (ST) targeting Signal Transducer and Activator of Transcription 3 (STAT3), which is overexpressed in many cancers and induceswhich contributes to tumorderived immune suppression, prior to vaccination, we observed enhancement of the survivinspecific response resulting in significant control of the primary tumor, reduction in lung metastases, and extension in survival. Thus, modulating tumor-derived immune suppression prior to therapeutic vaccination was sufficient to rescue the immune response and cause tumor regression. We then sought to apply the same approach to PDAC. We postulated, however, that although while PDAC is a prime candidate for Salmonella-based treatment due to its hypoxic nature [9,10], it is poorly immunogenic and many of the physical barriers, such as interstitial pressure generated by surrounding fibrosis, would prevent ST from penetrating into the hypoxic regions, thus requiring alternative intervention methods. In the PDAC stromal compartment, hyaluronic acid (HA) can be found at extremely high levels in the extracellular matrix (ECM) [11]. This results in a biophysical barrier that significantly reduces delivery of therapeutics to tumor cells. Our recent studies have shown that using PEGPH20, a PEGylated human recombinant PH20 hyalurodinase that depletes the HA found in the ECM of PDAC, helps to decrease interstitial and increase the permeability of the tumor to biological vectors such as attenuated ST [12]. Following PEGPH20 treatment in aggressive murine models of PDAC, we have found that administration of ST transformed with an shRNA plasmid specific to the immunosuppressive protein indoleamine 2,3-dioxygenase [13,14], results in enhanced ST colonization and intratumoral recruitment of PMN which are involved in the direct killing of surrounding tumor cells. The advantage of this therapeutic approach is that it induces innate immunity specifically within tumor tissue to suppress growth. This is beneficial in patients who are immune compromised; especially those who have been the recipients of chemotherapy treatments, as PMN have extremely rapid turnover rates. There is also evidence that the combination of PEGPH20/shIDO-ST treatment induces late anti-tumor CD8+ T cell responses critical for long-term tumor control. We have initiated studies to further characterize this response in hopes of determining its specific antigenic target and the role of PMN in its generation. While we have observed induction of potent innate and adaptive anti-tumor responses, we have not observed significant toxicity in pre-clinical cancer models, thus providing some evidence of safety. Further studies of efficacy and toxicity in patient-derived xenograft and humanized-IDO transgenic models are planned. In conclusion, we believe that PEGPH20/shIDO-ST represents a multi-pronged strategy that will potentially have greater efficacy than present chemotherapeutic options for PDAC and other solid tumors and is currently being developed for clinical application.
